# Infant SES as a Predictor of Personality—Is the Association Mediated by Intelligence?

**DOI:** 10.1371/journal.pone.0103846

**Published:** 2014-07-31

**Authors:** Trine Flensborg-Madsen, Erik Lykke Mortensen

**Affiliations:** 1 Unit of Medical Psychology, Institute of Public Health, University of Copenhagen, Copenhagen, Denmark; 2 Center for Healthy Aging, The Faculty of Health and Medical Sciences, University of Copenhagen, Copenhagen, Denmark; 3 Institute of Preventive Medicine, Bispebjerg and Frederiksberg Hospital, The Capital Region, Copenhagen, Denmark; Tilburg University, Netherlands

## Abstract

**Background:**

Although research into the continuity and change of personality traits during a lifespan has been fairly extensive, little research has been conducted on childhood predictors of adult personality.

**Purpose:**

We aimed to investigate the association between infant socioeconomic status (SES), and Eysenck personality traits in adulthood. An additional aim was to investigate whether intelligence and education may mediate this association.

**Methods:**

SES of 9125 children in the Copenhagen Perinatal Cohort was recorded at a 1-year examination. A subsample of this cohort, comprising 1182 individuals, participated in a follow-up at 20–34 years and was administered the Eysenck Personality Questionnaire (EPQ) which includes measures of neuroticism, extraversion, psychoticism and the so-called lie-scale. Associations of SES with each of the four personality traits were analysed by bivariate and partial correlations, and the mediating effects of intelligence and years of education were analysed.

**Results:**

Higher SES in infancy was associated with lower neuroticism (r = −0.06; p = 0.05), lower lie-scale scores (r = −0.11; p = 0.0002), and higher psychoticism (r = 0.09; p = 0.003). However, analyses of mediation revealed no direct effect of infant SES on any of the adult personality traits, but only indirect effects mediated by intelligence and years of education, with intelligence being the main mediating factor.

**Conclusion:**

Only weak associations were observed between infant SES and personality in young adulthood, and the observed associations were mediated by adult intelligence and educational level. Thus, factors associated with infant SES or family background appears to have weak direct effects on personality development.

## Introduction

Increasing evidence suggests that specific personality traits are related to numerous facets of human life, including subjective well-being [1], stress [2], psychopathology [3], morbidity [4] and longevity [5], [6]. However, although research into consequences of personality in addition to the continuity and change of personality traits during a lifespan [7] has been fairly extensive, research into possible early predictors of personality development, especially demographic factors such as socioeconomic status (SES), is limited.

With respect to *adult measures of SES* and personality, a recent Danish cross sectional study observed associations between occupational social class and personality: Neuroticism increased, whereas extraversion and conscientiousness decreased with lower social class [8]. In accordance with these results, an American study found low SES to be associated with higher neuroticism and agreeableness and with lower extraversion, openness, and conscientiousness [9], and a recent study from Scotland using a dichotomized measure of area deprivation found higher scores on neuroticism and psychoticism in the “most deprived group” compared to “the least deprived group” [10]. In spite of the studies on associations between adult measures of SES and personality, little research has focused on understanding these associations which may reflect causal effects of SES on personality, causal effects of personality on SES, and correlations with other factors which influence both SES and personality. While SES and personality may mutually influence each other in adult individuals, it is possible that the associations between SES and personality are best understood in a developmental perspective as factors associated with childhood or family SES may influence personality development and thus contribute to the adult associations. However, the possible effects of SES early in life on personality development have been little researched, and we have only been able to locate two relevant studies. These studies found that low childhood SES was associated with cynical hostility [11] and with high neuroticism, low conscientiousness, low extraversion and low openness in adulthood [12].

Strong associations are often observed between early life SES and measures of adult intelligence and educational level, and since these factors are also associated with measures of personality [13], there are reasons to believe that intelligence and education would play an important role in the association between infant/childhood SES and personality. Intelligence is often found to be more strongly associated with demographic factors than personality [14], [15], and cognitive ability and academic achievement have been found to correlate with certain personality traits [8], [13], [16]. Hence, these cognitive factors may possibly mediate associations between early life SES and adult personality. However, neither of the two studies investigating early life SES and adult personality incorporated analyses of possible mediation by cognitive factors such as intelligence or education, and they were both either cross-sectional or retrospective in design.

The primary aim of the present study was to conduct analyses of the prospective associations between infant socioeconomic status (SES) and adult personality as assessed by the Eysenck Personality Questionnaire. Based on the available literature, we hypothesized that low infant SES would be associated with especially high neuroticism, and we also hypothesized that intelligence and years of education might mediate this association.

## Materials and Methods

### Study population

The study objectives were investigated using data from the Copenhagen Perinatal Cohort (CPC) and from a follow-up study of this birth cohort, The Prenatal Development Project (PDP). The CPC was initially established with data on 8,949 mothers and their 9,125 consecutive deliveries at the University Hospital in Copenhagen during the period 1959–61. Information on demographic, socioeconomic, prenatal, and postnatal factors were recorded prospectively during pregnancy, at delivery, and at a 1-year examination [17]. The mothers were mainly residents in Copenhagen, but some were admitted on obstetrical complications or because of single mother status [18]. A total of 8,400 infants survived the first month after birth. A subsample from the CPC participated in an ongoing research program between 1982–1994 that focused on the developmental effects of prenatal and perinatal factors, in particular the effects of prenatal exposure to prescribed maternal medications. On the basis of perinatal records, 1575 potential subjects were invited to participate in the PDP between 1982 to 1994 [19]. The full test battery included a 2–4 hour home assessment by a social worker and an 8–11 hour psychological evaluation conducted at the Institute of Preventive Medicine [19],[20]. Several personality and cognitive tests were administered, including the Eysenck Personality Questionnaire (EPQ) [21], which was administered to 1182 participants (75% of those contacted), and the Danish version of the Wechsler Adult Intelligence Scale [22] which was administered to 1155 participants (73% of those contacted). Among the 1182 participants with EPQ data, 532 participants were prenatally exposed to medication (primarily hormones and barbiturates) while 650 were unexposed matched controls. According to Danish regulations the present analyses do not require approval by the scientific ethical committee system.

### Socioeconomic status

*Infant socioeconomic status* (SES) was measured at the 1-year examination and was based on the social grouping of the Centre International de l’Enfance [23], in which points, 0–5, are given according to 1) the occupation of the breadwinner, 2) the way in which the breadwinner earns his/her wages (public relief, daily wage, weekly wage, monthly salary and own business or capital; 3) the education of the breadwinner; 4) the character of the living accommodation (its size, the number of persons per room, its position, etc.). When the data were first computerized more than 40 years ago, the original 0–20 point scale was converted to a scale ranging from 0 to 8 (with 8 being the highest SES) [24]. In the present study, sample frequencies of the 8 SES categories were: n = 20, 107, 215, 205, 134, 143, 145, and 86 respectively.

### Adult follow-up

#### Eysenck Personality Questionnaire (EPQ)

The EPQ was published in 1975 [21], and the Danish version comprises 101 binary ‘yes’ or ‘no’ questions from which scores on the personality traits of neuroticism, extraversion, psychoticism and a lie-scale are derived [25].

#### Intelligence

The complete version of the original Wechsler’s Adult Intelligence Scale (WAIS) was administered [26]. From the 11 subtests three IQ scores are derived. The presented analyses are primarily based on the Full Scale IQ, but relevant correlations will also be presented for the Verbal and Performance IQ scales.

#### Years of education

The total number of years the respondent attended primary and secondary school, ranging from 5 to 13.

### Statistical analyses

Among the 1182 participants completing the EPQ, the missing data rate on infant SES was 10.7%. The participants without information on infant SES did not differ significantly from those with information with regard to personality and IQ, and they were excluded from the main analyses. Numbers, means, standard deviations and skewness/kurtosis for SES, outcome variables (Eysenck personality traits) and potential mediators (intelligence and years of education) are presented in Table 1 for men and women separately. The inter-correlations of all variables are presented in a correlation matrix (Table 2).

**Table 1:** Means and standard deviations for infant SES, personality traits and potential mediators.

STYDY VARIABLE	MEN				WOMEN			
	Skewness/Kurtosis	n	Mean	SD	Skewness/Kurtosis	n	Mean	SD
**Infant SES (1–8):**	0.16/−0.97	531	4.672	1.879	0.19/−1.08	524	4.674	1.886
**Personality traits:**								
** Neuroticism**	0.79/0.01	592	6.100	4.536	0.40/−0.60	590	9.368	5.346
** Extraversion**	−0.77/−0.01	592	15.184	4.148	–0.87/0.23	590	14.244	4.436
** Psychoticism**	0.41/−0.03	592	4.606	2.318	0.91/0.81	590	3.442	2.240
** Lie-scale**	0.52/−0.04	592	6.546	3.343	0.51/0.04	590	7.610	3.475
**Potential mediators:**								
** Adult IQ**	–0.35/0.05	584	102.195	16.632	–0.35/0.14	571	102.091	14.245
** (Verbal IQ)**	–0.36/−0.06	584	102.015	16.782	–0.25/0.08	571	102.853	14.062
** (Performance IQ)**	–0.22/−0.06	584	101.986	15.887	–0,48/0.58	571	100.743	14.952
** School years (offspring)**	0.00/−1.04	583	10.835	1.737	0.10/−1.26	570	10.979	1.600

**Table 2:** Correlation matrix (Pearson’s correlation).

	SES	Neuro-ticism	Extra-version	Psychoti-cism	Lie-scale	Full IQ	Verbal IQ	Perfor-mance IQ	School years
**SES**	1								
**Neuroti-cism**	–0.061*	1							
**Extraver-sion**	0.042	–0.354**	1						
**Psychoti-cism**	0.091*	0.014	0.087*	1					
**Lie-scale**	–0.114*	–0.003	–0.161**	–0.273**	1				
**Full IQ**	0.381**	–0.180**	0.035	0.065*	–0.201**	1			
**Verbal IQ**	0.429**	–0.178**	0.024	0.071	–0.207**	0.931**	1		
**Perfor-mance IQ**	0.241**	–0.141**	0.041	–0.042	–0.150**	0.874**	0.637**	1	
**School years**	0.484**	–0.118**	0.022	0.073*	–0.129**	0.607**	0.650**	0.419**	1

*<0.05.

**<0.0001.

**Figure 1:**
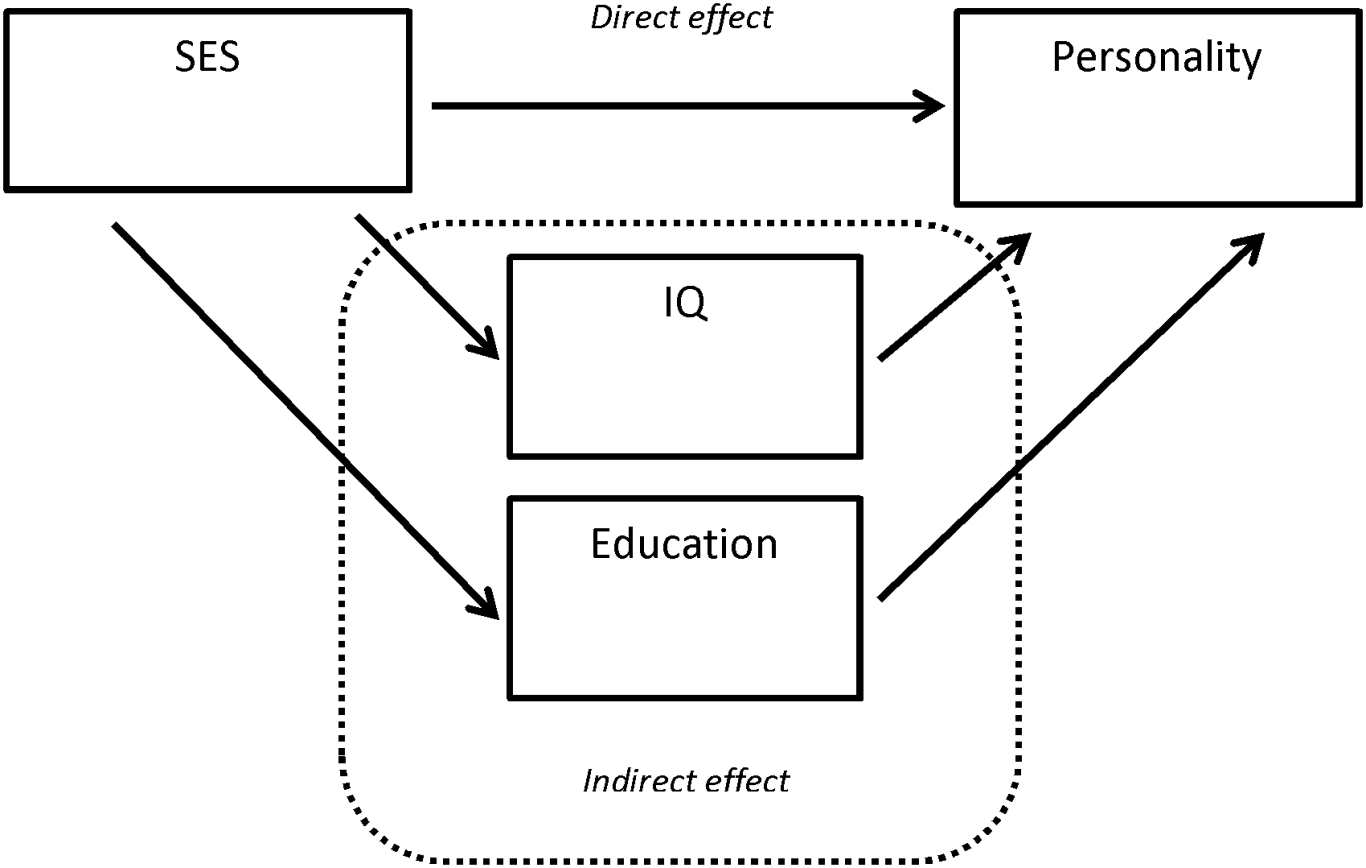
Overview of mediational effects.

The associations between SES and each of the four personality traits were estimated by bivariate and partial correlations (Table 3). For partial correlations, two models are presented: A model with gender and age at adult follow-up included as covariates and a model with gender, age at adult follow-up, and the potential mediators, intelligence and years of education, included (Table 3). To evaluate the influence of missing data, we used the structural equation modelling facilities of Stata 13 (StataCorp LP, USA) to repeat all correlations using full information maximum likelihood (FIML [27]). These analyses use all available information, including information on covariates and personality traits for participants without information on infant SES. The results for the fully adjusted FIML model are presented together with the corresponding model based on the subsample of participants with complete information on all variables (Table 3). The role of potential mediating factors were evaluated based on estimates of direct and indirect effects (Cf, Figure 1) and calculated by the Stata add-on procedure ‘binary mediation’ (Phil Ender, UCLA Academic Technology Service), with bootstrap standard errors used to calculate p-values. Gender and age at adult follow-up were included in the model (Table 4). All analyses presented in Table 4 were also conducted as FIML using the “sem” procedure of Stata (data not shown).

**Table 3:** Pearson correlations for infant SES according to Eysenck personality traits.

Eysenck personality trait:	N	Bivariate	Partial*	Partial**	Partial, FIML adjusted**
		r *(p)*	r *(p)*	r *(p)*	r *(p)*
**Neuroticism**	1055	–0.061 (0.046)	–0.085 (0.006)	–0.010 (0.762)	–0.009 (0.792)
**Extraversion**	1055	0.042 (0.173)	0.044 (0.155)	0.036 (0.245)	0.042 (0.237)
**Psychoticism**	1055	0.090 (0.003)	0.077 (0.014)	0.032 (0.313)	0.034 (0.319)
**Lie-scale**	1055	–0.114 (0.0002)	–0.131 (<0.0001)	–0.042 (0.184)	–0.042 (0.230)
					
**Intelligence**	1030	0.381 (<0.0001)	0.381 (<0.0001)	0.131 (<0.0001)	0.121 (<0.0001)
**Years of education**	1029	0.484 (<0.0001)	0.479 (<0.0001)	0.338 (<0.0001)	0.293 (<0.0001)

*Gender, age at adult follow-up.

**Gender, age at adult follow-up, intelligence, and years of education.

FIML: Full Information Maximum Likelihood.

**Table 4:** Indirect and direct effects of infant SES on EPQ personality traits^1^.

Variable	Neuroticism		Extraversion		Psychoticism		Lie Scale	
Effects of Parental SES:	*Effect*	*P (SE)*	*Effect*	*P (SE)*	*Effect*	*P (SE)*	*Effect*	*P (SE)*
**Indirect Effects**								
Offspring Education	–0.023	0.211 (0.018)	–0.002	0.936 (0.022)	0.026	0.163 (0.019)	–0.009	0.638 (0.020)
Offspring Intelligence	–0.052	0.014 (0.014)	0.005	0.725 (0.015)	0.017	0.234 (0.014)	–0.073	<0.001 (0.016)
Total Indirect Effect	–0.075	<0.001 (0.017)	0.003	0.853 (0.018)	0.043	0.010 (0.016)	–0.082	<0.001 (0.018)
**Direct Effect**	–0.010	0.741 (0.031)	0.042	0.256 (0.037)	0.035	0.301 (0.034)	–0.047	0.176 (0.034)
**Total Effect**	–0.085	0.003 (0.028)	0.045	0.170 (0.033)	0.078	0.006 (0.028)	–0.129	<0.001 (0.030)

1 Stata add-on procedure binary mediation was used to calculate effects. This procedure analyses both binary and continuous outcomes. Bootstrap standard errors are used to calculate p-values. Gender and age at adult follow-up are included in the model.

To evaluate the potential modifying effects of gender and age at adult follow-up, the interaction of both variables with infant SES was tested for all personality traits and revealed no significant interaction terms. The level of infant SES was significantly higher in the medication exposure group, but the interaction between prenatal medication exposure status and infant SES was tested and found non-significant for all personality traits (data not shown).

All statistical analyses (except for the mediational analyses in Table 4) were conducted by means of the statistical software package SAS 9.1.

## Results

Table 1 displays the number of observations, means, and standard deviations for SES, EPQ personality traits, and potential mediators. On the EPQ, standard deviations were largest for neuroticism (mean = 7.63, SD = 5.16), while it was smallest for psychoticism (mean = 4.04, SD = 2.36). Cronbach’s alpha values for neuroticism, extraversion, psychoticism and lie-scale were 0.84/0.87, 0.82/0.84, 0.43/0.48, and 0.74/0.74 for men and women respectively. The correlation matrix revealed significant correlations especially among SES, intelligence, and years of education. Among the EPQ traits, especially neuroticism and extraversion were significantly correlated (r = −0.35, p<0.0001). Correlation matrices for each sex were computed and showed generally similar correlations for men and women. Nevertheless, psychoticism was significantly associated with more covariates among men, while number of school years was significantly associated with more covariates among women (data not shown).

Results of the bivariate analyses in Table 3 showed that higher SES at birth was associated with lower neuroticism (r = –0.06; p = 0.05) and lie-scale score (r = –0.11; p = 0.0002) and higher psychoticism (r = 0.09, p = 0.003). Adjusted analyses including gender and age at adult follow-up did not change the estimates substantially, while the inclusion of intelligence and years of education reduced the partial correlations to non-significance. Both intelligence and years of education were significantly associated with infant SES in bivariate correlations (r = 0.38; p<0.0001 and r = 0.49; p<0.0001 respectively) as well as mutually adjusted partial correlations. The results of the FIML analyses incorporating all available information were essentially the same as those of the analyses based on subsamples with complete information on all relevant variables.

Estimates of indirect and direct effects of infant SES on EPQ personality traits (Table 4) showed no significant direct effects of infant SES when offspring intelligence and education were included in the analysis. The total indirect effect of the two factors was, however, significant for neuroticism (β = –0.07; p<0.0001), psychoticism (β = 0.04; p = 0.01), and lie-scale (β = –0.08; p<0.0001). Intelligence appeared to be the main mediating factor with significant indirect effects on both neuroticism (β = –0.05; p = 0.014) and lie-scale (β = –0.07; p<0.0001). All estimates in Table 3 were generally the same using FIML (data not shown).

## Discussion

The current study expands the life course epidemiological research of personality predictors by examining a well-validated measure of personality traits (EPQ) in relation to parental SES at infancy. Our main results were that there are only weak associations between infant SES and adult personality, and the observed significant associations between SES and neuroticism, lie-scale, and psychoticism were all found to reflect a mediating effect of intelligence (and to some extent years of education).

The main advantage of this study is the prospective design including real-time registration of infant SES. However, as in all observational studies, there may be unrecognized confounding factors associated with SES and personality. Gender, adult age at follow-up, intelligence, and years of education were included in the models. However, there may be other unobserved confounding or mediating factors in the associations. Factors associated with infant SES, intelligence, and personality in adulthood could be genetic factors or environmental factors related to the upbringing of children, such as attachment to the primary caregiver, early neglect, or reduced stimulation in the first years. These factors were not included in the analyses as appropriate data were not available, and therefore we do not know the impact of these factors on the associations at hand. Furthermore, only information on breadwinner’s SES was available, while independent SES information on both parents would have permitted more detailed analyses. The relevance of such analyses was demonstrated by Jonassaint et al. [12] who showed that mother’s and father’s education had different influences on the personality of the offspring. An anonymous reviewer has pointed out that the relatively small correlations between infant SES and adult personality might reflect response styles rather than true variation in adult personality dimensions. We find this a possible explanation of a correlation between infant SES and single adult personality dimension (e.g. a single association with the lie scale), but find it unlikely that response style can explain correlations with three of the four EPQ scales, including positive correlations with psychoticism and negative correlations with both neuroticism and the lie-scale. Finally, the data on intelligence and personality are cross-sectional which raises the issue of the direction of causality. In a developmental perspective it is likely that intelligence influences personality development, but also that personality to an extent influences development of intelligence. However, we have chosen to analyse intelligence as a mediator of the association between SES and personality because stable individual differences in intelligence tend to develop earlier than stable individual differences in personality [28] and because associations between SES and intelligence are usually found to be much stronger than associations between SES and personality [8], [15] (which was also the case in the present study).

In the present study we observed a correlation of 0.38 between infant parental SES and adult intelligence. This very substantial correlation is likely to reflect both common genetic variance and environmental influence on development of intelligence since evidence of genetic effects on both SES and intelligence have been observed in Danish Studies [29], [30]. Furthermore, the heritability of both intelligence and personality has been studied extensively and individual differences in both have been shown to be substantially influenced by genetic variance [31]. Twin research suggests that genetic variance in intelligence and personality partly overlaps [32]–[34] and both phenotypic and genotypic covariance between intelligence and personality have been observed [31], [35], suggesting that shared genetic variance is one important component in the association between intelligence and personality. Thus, associations among infant SES, intelligence and personality may reflect common genetic variance, and the fact that intelligence in this study appears to mediate the relationship between SES and personality does not imply that a causal pathway is purely environmental.

The most comparable findings from previous studies are: (1) The study by Jonassaint et al. [12] showing that parent’s education in childhood was associated with NEO PI-R personality traits; thus higher father’s education was associated with lower neuroticism, and higher extraversion, openness and conscientiousness, whereas higher mother’s education was significantly associated with higher extraversion and openness. (2) The study by Harper et al. [11] found that childhood socioeconomic position was associated with adult psychosocial functioning in terms of cynical hostility and hopelessness (the latter presumably related to the broad dimension of neuroticism). None of the studies, however, analysed intelligence and school years as potential mediators of the associations, and they were both retrospective, thereby increasing the risk of recall-bias, perhaps explaining the somewhat larger correlations (β = 0.15–0.20) [12].

In addition to the findings of Jonassaint et al. [12] our findings of higher infant SES being associated with lower neuroticism is in accordance with a recent large Danish cross sectional study that found lower social class (in adulthood) to be associated with higher neuroticism [8]. The trait of neuroticism, commonly described in terms of “anxious, depressed, guilt feelings, low self-esteem, tense, irrational, shy, moody, emotional” [2], has also in several studies been shown to be associated with lower intelligence, supporting that IQ is a main factor explaining the association between SES and neuroticism (although correlations have sometimes been interpreted in terms of the influence of personality on a cognitive test performance or the influence of intelligence on the interpretation of items in personality inventories [13]).

We did not find any significant associations between infant SES and extraversion in adulthood, which is not in accordance with the study by Jonassaint et al. [12] finding higher education of either parent to be associated with high extraversion in adulthood or with a Danish cross sectional study finding lower social class to be associated with decreased extraversion [8]. Correlations between intelligence and extraversion have generally been varying in studies investigating the issue [13], [31] with results differing from positive to negative associations.

The validity and meaning of the trait psychoticism, as measured by Eysenck, has been subject to much discussion and has been criticised for its low internal consistency (i.e. low inter-item correlations) and a weak factor structure [36]–[38]. Psychoticism has been interpreted as being either a sub dimension of or a blend of the traits *agreeableness* and *conscientiousness* [39]. Howarth [38] suggested “that users of the P scale might obtain results varying widely from study to study according to (among other independent variables) the composition of the study sample which might favour one or other aspect of this composite at present called ‘psychoticism’ ”. Our study found a relationship between high infant SES and high psychoticism, while the mediation analyses found this association to be mediated by the total indirect effects of intelligence and education. Generally, studies indicate that psychoticism is negatively related to academic achievement but positively related to openness [13], which has been found to correlate with intelligence [13].

Lie scales were originally introduced into personality measures in order to detect the “faking good” of scores on other scales [40]. It has, however, been suggested that lie scales in general should be interpreted as measuring a personality dimension in its own right [41], [42]. According to Eysenck & Eysenck [43], the lie scale included in the Eysenck Personality Questionnaire (EPQ) permits lying to be diagnosed, but the unitary nature of the Eysenckian lie scale has been questioned, and more than one distinct personality component has been suggested [44]–[46]. According to some researchers, the dimension is best characterized as *social acquiescence or conformity* [47], [48], and according to others as (lack of) *self-insight* [44], [49]–[51]. Our findings of higher infant SES being associated with lower lie-scale scores cannot directly be compared to other studies as the subject, to our knowledge, has not yet been investigated elsewhere. Nevertheless, three different studies found significant negative associations between birth weight (a known positive correlate of both infant SES and intelligence) and lie-scale scores in adulthood [52]–[54], which indirectly supports our findings of intelligence being a mediating factor in the association.

Life course epidemiology has consistently shown a significant correlation between low SES in childhood and poor health in adulthood [55]–[60], and it has been hypothesized that early emerging personality may mediate the effect of childhood SES on adult health. This mediating effect of personality has been demonstrated in studies indicating that SES influences health through sense of control [61], [62] and ‘reserve capacity’ (defined by combined measures of optimism/pessimism, sense of control, self-esteem, and social support to measure) [63]. In addition, “at risk” personality traits have consistently been shown to be associated with risky behaviours and poor health [64]–[71], thereby further supporting the hypothesis of personality being a mediating factor in the known association between low SES and poor health. However, intelligence typically shows stronger associations with both SES and health outcomes than measures of personality [15], [72], [73], and our results suggest that intelligence, rather than personality, may be the important factor linking SES to health.

## Conclusion

This first prospective study of infant SES and personality observed only weak associations between parental SES in infancy and adult personality. Whereas small significant associations were found between high SES and lower neuroticism, higher psychoticism and lower lie-scale scores – these associations were all found to be mediated by intelligence and number of school years. As this is the first study to investigate this issue, more research should be carried out in the area to elucidate and corroborate the present findings.

Although we found several statistically significant associations, the obtained correlations are relatively small and of limited practical significance. However, the results might not generalize to societies in which disparities in SES are considerably larger than in Denmark and higher correlations between SES and personality might be observed. In addition, our results can be of importance in health research, where personality has previously been suggested to be an important factor linking childhood SES to poor health. Based on the present findings we suggest that intelligence, rather than personality should be the primary focus in such studies.

## References

[1] Lucas RE, Diener E (2008) Personality and Subjective Well-being. In: John OP, Robins RW, Pervin LA, editors. Handbook of personality. New York: The Guilford Press.

[2] Matthews G, Deary IJ, Whiteman MC (2009) Personality traits. United Kingdom, Cambridge: University Press.

[3] Widiger TA, Smith GT (2008) Personality and Psychopathology. In: John OP, Robins RW, Pervin LA, editors. Handbook of personality. New York: The Guilford Press.

[4] Antoniou EE, Dutta A, Langa KM, Melzer D, Llewellyn D (2013) Personality Profile of the Children of Long-Lived Parents. Journals of Gerontology Series B-Psychological Sciences and Social Sciences 68: 730–738. 10.1093/geronb/gbt003 PMC5975667 23419869

[5] Andersen SL, Sun JX, Sebastiani P, Huntly J, Gass JD, et al (2013) Personality Factors in the Long Life Family Study. Journals of Gerontology Series B-Psychological Sciences and Social Sciences 68: 739–749. 10.1093/geronb/gbs117 PMC3744045 23275497

[6] Terracciano A, Lockenhoff CE, Zonderman AB, Ferrucci L, Costa PT (2008) Personality predictors of longevity: Activity, emotional stability, and conscientiousness. Psychosom Med 70: 621–627. 18596250 10.1097/PSY.0b013e31817b9371 PMC2505356

[7] Roberts BW, Wood D, Caspi A (2008) The Development of Personality Traits in Adulthood. In: John OP, Robins RW, Pervin LA, editors. Handbook of Personality. New York: The Guilford Press.

[8] Mortensen EL, Flensborg-Madsen T, Molbo D, Christensen U, Osler M, et al (2013) Personality in late midlife. Associations with demographic factors and cognitive ability. Journal of Aging and Health 26: 21–36. 10.1177/0898264313519317 24584258

[9] Chapman BP, Fiscella K, Kawachi I, Duberstein PR (2010) Personality, socioeconomic status, and all-cause mortality in the United States. Am J Epidemiol 171: 83–92. 19965888 10.1093/aje/kwp323 PMC2800299

[10] Millar K, Lloyd SM, McLean JS, Batty GD, Burns H, et al (2013) Personality, socio-economic status and inflammation: cross-sectional, population-based study. Plos One 8: e58256. 23516457 10.1371/journal.pone.0058256 PMC3596406

[11] Harper S, Lynch J, Hsu WL, Everson SA, Hillemeier MM, et al (2002) Life course socioeconomic conditions and adult psychosocial functioning. Int J Epidemiol 31: 395–403. 11980802

[12] Jonassaint CR, Siegler IC, Barefoot JC, Edwards CL, Williams RB (2011) Low Life Course Socioeconomic Status (SES) is Associated with Negative NEO PI-R Personality Patterns. International Journal of Behavioral Medicine 18: 13–21. 20012811 10.1007/s12529-009-9069-x PMC3634575

[13] Chamorro-Premuzic T, Furnham A (2005) Personality and Intellectual Competence. New Jersey: Lawrence Erlbaum Associates, Inc., Publishers.

[14] Calvin CM, Deary IJ, Fenton C, Roberts BA, Der G, et al (2011) Intelligence in youth and all-cause-mortality: systematic review with meta-analysis. Int J Epidemiol 40: 626–644. 21037248 10.1093/ije/dyq190 PMC3147066

[15] Gottfredson LS (2004) Intelligence: Is it the epidemiologists’ elusive “Fundamental cause” of social class inequalities in health? J Pers Soc Psychol 86: 174–199. 14717635 10.1037/0022-3514.86.1.174

[16] DeYoung CG (2011) Intelligence and personality. In: Sternberg RJ, Kaufman SB, editors. Cambridge handbook of intelligence. New York: Cambridge University Press. 711–737.

[17] Zachau-Christiansen B, Ross EM (1975) Babies: human development during the first year. John Wiley.

[18] Villumsen AL (1970) Environmental factors in congenital malformaitons: a prospective cohort study of 9,006 human pregnancies. Copenhagen: FADL’s Forlag.

[19] Reinisch JM, Mortensen EL, Sanders SA (1993) The Prenatal Development Project. Acta Psychiatr Scand 87: 54–61. 10.1111/j.1600-0447.1993.tb05361.x 8452055

[20] Mortensen EL, Sorensen HJ, Jensen HH, Reinisch JM, Mednick SA (2005) IQ and mental disorder in young men. Br J Psychiatry 187: 407–415. 16260814 10.1192/bjp.187.5.407

[21] Eysenck HJ, Eysenck SBG (1975) Manual of the Eysenck Personality Questionnaire. Sevenoaks, Kent: Hodder and Stoughton Educational.

[22] Wechsler D (1958) The measurement and appraisal of adult intelligence. The Williams & Wilkins Company.

[23] Graffar M (1960) Social study of samples. Mod Probl Pädiat 5: 30–42.

[24] Zachau-Christiansen B (1972) The influence of Prenatal and Perinatal Factors on Development During the First Year of Life. Helsingør: Poul A. Andersens Forlag.

[25] Hansen HS, Mortensen EL (2004) Dokumentation for den danske udgave af NEO-PI-R Kort version. [Danish]. Copenhagen: Psykologisk Forlag A/S. 86 p.

[26] Wechsler D (2012) The measurement and appraisal of adult intelligence.

[27] Graham JW (2009) Missing Data Analysis: Making It Work in the Real World. Annu Rev Psychol 60: 549–576. 18652544 10.1146/annurev.psych.58.110405.085530

[28] Mackintosh NJ (2011) IQ and human intelligence. New York: Oxford University Press.

[29] Teasdale TW, Owen DR (1984) Heredity and familial environment in intelligence and educational level–a sibling study. Nature 309: 620–622. 6728020 10.1038/309620a0

[30] Teasdale TW (1985) Familial Influences in Social Class, Educational Level and Intelligence. [dissertation]. Malmø: Gleerup.

[31] Pincombe JL, Luciano M, Martin NG, Wright MJ (2007) Heritability of NEO PI-R extraversion facets and their relationship with IQ. Twin Research and Human Genetics 10: 462–469. 17564504 10.1375/twin.10.3.462

[32] Ackerman PL, Heggestad ED (1997) Intelligence, personality, and interests: Evidence for overlapping traits. Psychol Bull 121: 219–245. 9100487 10.1037/0033-2909.121.2.219

[33] Harris JA, Vernon PA, Jang KL (1996) A multivariate genetic analysis of personality and intelligence. Behav Genet 26: 587.

[34] Luciano M, Wainwright MA, Wright MJ, Martin NG (2006) The heritability of conscientiousness facets and their relationship to IQ and academic achievement. Personality and Individual Differences 40: 1189–1199.

[35] Harris JA, Vernon PA, Olson JM, Jang KL (1999) Self-rated personality and intelligence: a multivariate genetic analysis. European Journal of Personality 13: 121–128.

[36] Eysenck SBG, Eysenck HJ, Barrett P (1985) A Revised Version of the Psychoticism Scale. Personality and Individual Differences 6: 21–29.

[37] Ferrando PJ (2008) The impact of social desirability bias on the EPQ-R item scores: An item response theory analysis. Personality and Individual Differences 44: 1784–1794.

[38] Howarth E (1986) What Does Eysenck Psychoticism Scale Really Measure. Br J Psychol 77: 223–227. 3730728 10.1111/j.2044-8295.1986.tb01996.x

[39] Heaven PC, Ciarrochi J, Leeson P, Barkus E (2013) Agreeableness, conscientiousness, and psychoticism: distinctive influences of three personality dimensions in adolescence. Br J Psychol 104: 481–494. 24094279 10.1111/bjop.12002

[40] O’Donovan D (1969) An historical review of the lie scale: with particular reference to the Maudsley Personality Inventory. Research in Psychology 3: 13–19.

[41] Furnham A (1986) Response Bias, Social Desirability and Dissimulation. Personality and Individual Differences 7: 385–400.

[42] McCrae RR, Costa PT (1983) Social Desirability Scales - More Substance Than Style. J Consult Clin Psychol 51: 882–888.

[43] Eysenck HJ, Eysenck SBG (1976) Psychoticism as a dimension of personality. London: Hodder and Stoughton.

[44] Francis LJ (1991) The Dual Nature of the Epq Lie Scale Among College-Students in England. Personality and Individual Differences 12: 1255–1260.

[45] Francis LJ, Brown LB, Pearson PR (1991) The Dual Nature of the Epq Lie Scale Among University-Students in Australia. Personality and Individual Differences 12: 989–991.

[46] Pearson PR, Francis LJ (1989) The Dual Nature of the Eysenckian Lie Scales - Are Religious Adolescents More Truthful. Personality and Individual Differences 10: 1041–1048.

[47] Birenbaum M, Montag I (1989) Style and Substance in Social Desirability Scales. European Journal of Personality 3: 47–59.

[48] Massey A (1980) The Eysenck Personality-Inventory Lie Scale - Lack of Insight Or. Irish Journal of Psychology 4: 172–174.

[49] Crookes TG, Buckley SJ (1976) Lie Score and Insight. Irish Journal of Psychology 3: 134–136.

[50] Francis LJ, Pearson PR, Kay WK (1983) Are Religious Children Bigger Liars. Psychol Rep 52: 551–554.

[51] Kirton M (1977) Characteristics of High Lie Scorers. Psychol Rep 40: 279–280. 840983 10.2466/pr0.1977.40.1.279

[52] Allin M, Rooney M, Cuddy M, Wyatt J, Walshe M, et al (2006) Personality in young adults who are born preterm. Pediatrics 117: 309–316. 16452348 10.1542/peds.2005-0539

[53] Flensborg-Madsen T, Revsbech R, Sørensen HJ, Mortensen EL (2014) An association of adult personality with prenatal and arly postnatal growth: the EPQ lie-scale. BMC Psychology 2: 8. 25566381 10.1186/2050-7283-2-8 PMC4270018

[54] Schmidt LA, Miskovic V, Boyle MH, Saigal S (2008) Shyness and timidity in young adults who were born at extremely low birth weight. Pediatrics 122: E181–E187. 18595963 10.1542/peds.2007-3747

[55] Frank JW, Cohen R, Yen I, Balfour J, Smith M (2003) Socioeconomic gradients in health status over 29 years of follow-up after midlife: the Alameda county study. Soc Sci Med 57: 2305–2323. 14572839 10.1016/j.socscimed.2003.08.003

[56] Power C, Matthews S, Manor O (1996) Inequalities in self rated health in the 1958 birth cohort: Lifetime social circumstances or social mobility? Br Med J 313: 449–453. 8776310 10.1136/bmj.313.7055.449 PMC2351851

[57] Smith GD, Hart C, Blane D, Gillis C, Hawthorne V (1997) Lifetime socioeconomic position and mortality: Prospective observational study. Br Med J 314: 547–552. 9055712 10.1136/bmj.314.7080.547 PMC2126019

[58] Smith GD, Hart C, Blane D, Hole D (1998) Adverse socioeconomic conditions in childhood and cause specific adult mortality: prospective observational study. Br Med J 316: 1631–1635. 9603744 10.1136/bmj.316.7145.1631 PMC28561

[59] Vagero D, Leon D (1994) Effect of Social-Class in Childhood and Adulthood on Adult Mortality. Lancet 343: 1224–1225. 10.1016/s0140-6736(94)92432-5 7909885

[60] Wamala SP, Lynch J, Kaplan GA (2001) Women’s exposure to early and later life socioeconomic disadvantage and coronary heart disease risk: the Stockholm Female Coronary Risk Study. Int J Epidemiol 30: 275–284. 11369727 10.1093/ije/30.2.275

[61] Bosma H, Schrijvers C, Mackenbach JP (1999) Socioeconomic inequalities in mortality and importance of perceived control: cohort study. Br Med J 319: 1469–1470. 10582929 10.1136/bmj.319.7223.1469 PMC28291

[62] Bosma H, Van Jaarsveld CHM, Tuinstra J, Sanderman R, Ranchor AV, et al (2005) Low control beliefs, classical coronary risk factors, and socioeconomic differences in heart disease in older persons. Social Science & Medicine 60: 737–745. 15571892 10.1016/j.socscimed.2004.06.018

[63] Matthews KA, Raikkonen K, Gallo L, Kuller LH (2008) Association between socioeconomic status and metabolic syndrome in women: Testing the reserve capacity model. Health Psychol 27: 576–583. 18823184 10.1037/0278-6133.27.5.576 PMC2880509

[64] Jonassaint CR, Boyle SH, Williams RB, Mark DB, Siegler IC, et al (2007) Facets of openness predict mortality in patients with cardiac disease. Psychosom Med 69: 319–322. 17510289 10.1097/PSY.0b013e318052e27d

[65] Sutin AR, Terracciano A, Deiana B, Naitza S, Ferrucci L, et al (2010) High Neuroticism and low Conscientiousness are associated with interleukin-6. Psychol Med 40: 1485–1493. 19995479 10.1017/S0033291709992029 PMC2933046

[66] Terracciano A, Costa PT Jr (2004) Smoking and the Five-Factor Model of personality. Addiction 99: 472–481. 15049747 10.1111/j.1360-0443.2004.00687.x PMC2376761

[67] Bernard NS, Dollinger SJ, Ramaniah NV (2002) Applying the big five personality factors to the impostor phenomenon. J Pers Assess 78: 321–333. 12067196 10.1207/S15327752JPA7802_07

[68] Wilson RS, de Leon CFM, Bienias JL, Evans DA, Bennett DA (2004) Personality and mortality in old age. Journals of Gerontology Series B-Psychological Sciences and Social Sciences 59: 110–116. 10.1093/geronb/59.3.p110 15118013

[69] Jokela M, Batty GD, Nyberg ST, Virtanen M, Nabi H, et al. (2013) Personality and All-Cause Mortality: Individual-Participant Meta-Analysis of 3,947 Deaths in 76,150 Adults. Am J Epidemiol. 10.1093/aje/kwt170 PMC3755650 23911610

[70] Jokela M, Hintsanen M, Hakulinen C, Batty GD, Nabi H, et al (2013) Association of personality with the development and persistence of obesity: a meta-analysis based on individual-participant data. Obes Rev 14: 315–323. 23176713 10.1111/obr.12007 PMC3717171

[71] Smith TW, MacKenzie J (2006) Personality and risk of physical illness. Annu Rev Clin Psychol 2: 435–467. 17716078 10.1146/annurev.clinpsy.2.022305.095257

[72] Batty GD, Deary IJ, Gottfredson LS (2007) Premorbid (early life) IQ and later mortality risk: Systematic review. Ann Epidemiol 17: 278–288. 17174570 10.1016/j.annepidem.2006.07.010

[73] Batty GD, Shipley MJ, Gale CR, Mortensen LH, Deary IJ (2008) Does IQ predict total and cardiovascular disease mortality as strongly as other risk factors? Comparison of effect estimates using the Vietnam Experience Study. Heart 94: 1541–1544. 18801778 10.1136/hrt.2008.149567 PMC2602751

